# Impact of anticancer therapy on the quality of life of Sudanese patients with breast cancer at Khartoum oncology hospital

**DOI:** 10.1186/s12905-022-02041-0

**Published:** 2022-11-14

**Authors:** Mawada Aldaak, Hayat M. Suliman, Elsadig Elgailany Abd-Elgadir, Iman Hassan Abdoon

**Affiliations:** 1 Clinical Pharmacy Program, Faculty of Pharmacy, University of Khartoum, Khartoum, Sudan; 2 Department of Pharmacology, Faculty of Pharmacy, University of Khartoum, Qassr Street, Khartoum, Sudan; 3 Consultant of Medical Oncology, Khartoum Oncology Hospital, Khartoum, Sudan

**Keywords:** Quality of life, Breast cancer therapy, Breast cancer, Predictors

## Abstract

**Background:**

Chemotherapy-related toxicity affects the quality of life (QOL) of patients with cancer. Measuring the QOL in breast cancer (BC) patients has been the focus of clinical practices and research in recent decades. This study aimed to assess the impact of BC medications on QOL of Sudanese patients with BC.

**Methods:**

A cross-sectional study was conducted in Khartoum Oncology Hospital, Sudan, from November 2020 to March 2021. All patients diagnosed with BC were included in the study. QOL was assessed using the European Organization for Research and Treatment of Cancer quality of life (EORTC QLQ-C-30) and breast cancer supplementary module (QLQ-BR23). ANOVA, independent t-test and logistic regression analysis were used to assess the association between variables.

**Results:**

Two hundred patients were enrolled in the study, with a mean age of 50 ± 11.7 years. 52.5% of the patients were on a conventional therapy whereas 40.5% and 7% received hormonal and HER2-targeted therapies, respectively. In QLQ-C30 scale, the global health-QOL status score was (53.2 ± 1.9), with 54.0% of patients having poor global health-QOL status. In the functional scale, the cognitive functioning was the highest score (80.7 ± 1.8). In QLQ-C30 symptom scale, the most distressing issue was financial difficulties (63.7 ± 2.9). In QLQ-BR23 scale, body image scored the worse functioning (47.7 ± 2.7), with 54.5% of patients having poor QOL. In QLQ-BR23 symptoms scale, “being upset by hair loss” was the highest disturbing symptom (62.1 ± 3.3), with 68.6% of patients having poor QOL. Global health status (*P* = 0.000), social (*P* = 0.000), emotional (*P* = 0.002) and role functioning (*P* = 0.000) were significantly higher in patients taking HER2-targeted or hormonal therapy compared to conventional therapy. The level of symptomatology was significantly low in patients taking HER2-targeted therapy or hormonal therapy (*P* = 0.000) than those on conventional therapy. Hormonal (OR = 3.7, *p* = 0.01) and HER2-targeted therapies (OR = 10.2, *p* = 0.04 ) were positive predictors of QOL.

**Conclusion:**

BC survivors in Sudan had a low QOL/global health status. Hormonal and HER2-targeted therapies were predictors of good QOL.

**Supplementary Information:**

The online version contains supplementary material available at 10.1186/s12905-022-02041-0.

## Background

Cancer is one of the major health problems that cause death worldwide [1, 2]. It remains a low priority for public health in Africa in general and in Sudan in particular [3]. However, WHO has developed global action plan for the prevention and control of non-communicable diseases, including cancer [4]. In Sudan, cancer is the third killer disease after malaria and viral pneumonia [5]. An epidemiological study in East Africa region, including Sudan, revealed that breast cancer (BC) is common amongst females [6]. Breast and cervical cancer account for about 50% of all cancers in Sudanese women. However, the incidence and prevalence of cancer were not determined in Sudan due to the absence of cancer registry program.

According to the expression of biomarkers (e.g., ER, PR, and HER2, and Ki67 proliferative protein), BC is categorized to major tumor subtypes: Luminal A, luminal B, HER2-enriched and triple negative types. Luminal A is a tumor with ER-positive, PR-positive, and low level of Ki67 protein. It has a good prognosis. Luminal B tumor is ER-positive, with high level of Ki67 protein and poor prognosis. HER2-enriched BC is HER2-positive, ER-negative and PR-negative, and it has a worse prognosis. The triple negative BC (TNBC) is a tumor with ER-negative, PR-negative, and HER2-negative. It tends to grow faster than the luminal cancers, with high degree of recurrence and metastasis [7, 8].

BC treatment modalities include breast surgery, radiotherapy and systemic therapies (endocrine therapy, chemotherapy, targeted therapy, and immunotherapy) [9, 10]. The decision on systemic treatment is based on several clinicopathological features such as ER, PR, and HER-2 status as well as the stage of cancer, tumor size, nodal involvement, menopausal status, age and health status of the patient [7]. In patients with TNBC, the conventional chemotherapy remains the standard treatment approach [11]. Endocrine therapy is recommended for HR (hormone receptor) positive patients. Tamoxifen (ER antagonist) is the standard drug for ER-positive patients, and it is used in both premenopausal and postmenopausal women whereas aromatase inhibitors (anastrozole, and letrozole) are used in postmenopausal women, especially with advanced BC [12]. HER2-directed therapy (e.g., Trastuzumab) is approved for the treatment of HER2-positive BC, and it may be safely combined with radiotherapy, chemotherapy, and endocrine therapy [11, 13].

In Sudan, the common chemotherapy regimens used for BC management include: CMF: (Cyclophosphamide, Methotrexate, 5-Fluorouracil), FAC (5- Fluorouracil, Doxorubicin, Cyclophosphamide), FEC (5-Fluorouracil, Epirubicin, Cyclophosphamide) and DAC (Docetaxel, Doxorubicin, Cyclophosphamide). Endocrine therapies (Tamoxifen or aromatase inhibitors) are prescribed to HR positive patients.

BC therapies have a detrimental impact on women's physical and emotional well-being due to adverse treatment outcomes [14]. Females with BC who are exposed to a variety of therapy may experience physical, mental and psychological distress associated with poorer body image and sexual problems [15]. In reality, women who suffer a loss of physical integrity show sudden changes in social relations [16, 17]. All these factors may negatively impact the quality of life (QOL) of BC patients [18].

Evaluation of QOL of BC patients is useful for monitoring person's health and well-being, and it is increasingly being used as an important outcome in clinical practice [19, 20]. Therefore, this study aimed to assess QOL of BC patients in Khartoum Oncology Hospital, Sudan, and to investigate the impact of medications on their QOL.

## Methods

### Study design

This is a descriptive, cross-sectional, hospital-based study.

### Study area and study period

The study was conducted in Khartoum Oncology Hospital in Khartoum city, Sudan from November 2020 to March 2021. Khartoum oncology hospital is the first center in Sudan for the diagnosis and treatment of cancer. It provides medical services such as radiotherapy, chemotherapy, laboratory and radiological diagnostic services specialized in oncology. This hospital provides medical services for more than 8000 new cases annually, in addition to the regular follow-up that exceeding 660,000 cases.

### Study population

Study population were BC patients attending Khartoum oncology hospital during the study period.

#### Inclusion criteria

• Female and male patients clearly diagnosed with BC
• Only Sudanese patients were included in the current study to negate the impact of culture differences on the research results.

#### Exclusion criteria

• Terminally ill patients who were unable to communicate
• Patients with incomplete medical records (e.g., anticancer medications) were excluded.
• Patients who were unwilling to participate

### Sample size and sampling technique

A total coverage sampling, during the study period, was used in the current study. Two hundred breast cancer patients were enrolled according to the inclusion and exclusion criteria.

### Data collection tools

The data was collected from BC patients attending Khartoum oncology hospital, using data collection sheets and self-administered questionnaire. The patients directly filled in the questionnaire after obtaining inform consent. Clinical data were extracted from the patients’ medical records using data collection sheet. Precautions for protections from COVID 19 were followed during data collection. The European Organization for Research and Treatment of Cancer Quality of Life (EORTC QLQ-C30, v.3.0) questionnaire and the BC supplementary module (EORTC QLQ-BR23) were used to evaluate the QOL of BC patients. EORTC QLQ-C30 is a validated and reliable questionnaire that had been translated and validated in several languages. A validated Arabic version “EORTC QLQ-C30 Specific Arabic Version” was used to assess patients’ QOL [21]. The questionnaire consisted of three parts: demographic and clinical characteristics of the patients; quality of life of cancer patients and a specific breast cancer module. EORTC QLQ-C30 composed of 30 questions including one global health scale (GHS), five functional scales (physical, role, emotional, cognitive and social functioning) and nine symptoms scale (fatigue, nausea/vomiting, pain, dyspnea, insomnia, appetite loss, constipation, diarrhea and financial difficulties). A specific BC supplemental module (EORTC QLQ-BR23) comprised of 23 items that designed for measuring four functional scales (body image, sexual functioning, sexual enjoyment, future perspective) and four symptoms scales (systemic therapy side-effects, breast symptoms, arm symptoms, being upset by hair loss) [22]. EORTC QLQ-C30 is a 4-point Likert scale rated from 1 to 4 (1 = Very much, 2 = Quite a bit, 3 = A little, 4 = Not at all) with exception of the 7-point questions of the global health status. All patients’ responses were evaluated and scored using a scoring manual which was supplementary provided with the questionnaire. All of the raw scores were linearly transformed to scores ranging from 0 to 100. A high score for global and functional scales represents high levels of functioning/QOL while high score for symptoms scale represents a high level of symptomatology/problems and less functioning/QOL [23]. For functional and global health status, QOL score was dichotomized into good (score: ˃ 50) and poor QOL (score: ≤ 50). For symptoms scale, a score ≤ 50 represents good QOL whereas a score ˃ 50 represents poor QOL.

### Selection of breast cancer therapy in Khartoum oncology hospital

In Khartoum oncology hospital, treatment options for BC patients are individualized. The selection of the appropriate therapy is determined according to shared decision-making between the physician and patient based on many factors including tumor’s molecular subtype (ER, PR and HER2 assessment), stage of cancer, tumor size, nodal involvement, metastases, as well as age, menopausal status, general health, and financial status of the patient.

Systemic therapies include chemotherapy, hormonal therapy, and HER2-targeted therapy. These drugs can be administered alone, or given in multiple-drug regimens. Adjuvant therapy (after surgery) is generally recommended for patients at high risk of recurrence (micrometastatic disease), and neoadjuvant therapy (before surgery) is frequently given to patients with locally advanced breast cancer (e.g., stage III, or tumors larger than 5 cm).

There are two types of surgery in Khartoum oncology hospital: Modified radical mastectomy (MRM) and breast-conserving surgery (BCS). MRM is a procedure that involves removal of the entire breast including the skin, breast tissue, areola, and nipple as well as most of the axillary (armpit) lymph nodes. In BCS, the cancerous tissues and a small margin of surrounding normal tissues were removed. After BCS, adjuvant radiotherapy may be carried out.

### Data statistical analysis

Data were analyzed using the statistical package SPSS version 20. Simple descriptive statistics such as frequency and percentage were used to describe the distribution of participants. For continuous data, all variables were tested using ANOVA and independent t-test. To identify the predictors of QOL, multivariable logistic regression analysis was used to describe the relationship between the dependent (QOL scores) and independent variables (e.g., demographic/clinical characteristics, medications). *P*-value was set at < 0.05 and was considered statistically significant.

## Results

### Socio-demographic characteristics of the study sample

A total of 200 respondents participated in the study. Most participants (78%) were over 40 years old, with a mean age of 50 ± 11.7 years. 29% of patients had high school certificate and 70% were married. Most participants (80%) were unemployed and resided outside Khartoum state (57.5%) (Table 1).

**Table 1:** Socio-demographic characteristics of breast cancer patients at Khartoum Oncology Hospital

Variables	Frequency	Percent
Age (years)		
18–30	9	4.5
31–40	35	17.5
41–50	73	36.5
51–60	50	25.0
> 60	33	16.5
Education		
Illiterate	54	27.0
Primary school	43	21.5
High school	58	29.0
University	45	22.5
Residence		
Outside Khartoum state	115	57.5
Khartoum state	85	42.5
Marital status		
Married	140	70.0
Single	45	22.5
Divorced	8	4.0
Widow	7	3.5
Status		
Unemployed	160	80.0
Employed	40	20.0

### Clinical characteristics of breast cancer patients at Khartoum Oncology Hospital

Out of 200 BC patients, 43.5% had stage IIB BC and received 4 to 6 cycles of chemotherapy. 28.5% of patients had comorbid diseases, with 11% having diabetes mellitus. 78.5% of patients underwent surgery, and 52% received neoadjuvant therapy before surgery. 91.1% of the surgical patients were subjected to modified radical mastectomy, and only 8.9% underwent breast conserving surgery (BCS). After surgery, the patient stayed at the hospital for 3 to 5 days. Based on post-operative follow up visits, the patients attended to the hospital weekly for three weeks. 21 days after surgery, the patients were referred to the chemotherapy department (Table 2).

**Table 2:** Medical history and clinical characteristics of breast cancer patients at Khartoum Oncology Hospital

Variables	Frequency	Percent
Chemotherapy cycles		
1–3 cycles	48	24.0
4–6 cycles	87	43.5
> 6 cycles	45	22.5
Not reported	20	10.0
Stage of tumor		
Stage 1	14	7.0
Stage 11A	34	17.0
Stage 11B	87	43.5
Stage 111A	22	11.0
Stage 111*	43	21.5
Comorbidities		
Hypertension	20	10.0
Diabetes Mellitus	22	11.0
Hypertension + Diabetes Mellitus	6	3.0
Heart disease and/or osteoarthritis	4	2.0
Others	5	2.5
None	143	71.5
Surgery (*n* = 157)	157	78.5
Modified radical mastectomy	143	91.1
Breast-conserving surgery (BCS)	14	8.9

Stage 111*: tumor size larger than stage II and spreads to more than one lymph node and/or tissue around the breast or breast bone

### Anticancer medications for breast cancer patients at Khartoum Oncology Hospital

**Fig. 1:**
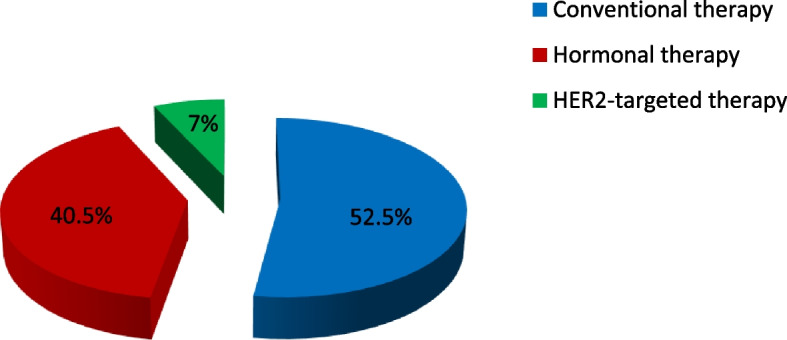
Types of medications for breast cancer patients in Khartoum Oncology Hospital

More than half of the patients (52.5%) were on conventional therapy, with 17.5% receiving (cyclophosphamide + 5-FU) in combination with epirubicin followed by docetaxel. Hormonal therapy was prescribed for 40.5% of the patients, with 15.5% taking tamoxifen. Only 7% of patients received HER2-targeted therapy. Trastuzumab was prescribed for 4.5% of patients with HER2-positive BC, and 2.5% received trastuzumab plus tamoxifen or letrozole (Table 3 and Fig. 1).

**Table 3:** Anticancer medications for breast cancer patients at Khartoum Oncology Hospital

Medications	Frequency	Percent
**Conventional therapy**		
(Cyclophosphamide/5-FU) + Doxorubicin or Doxorubicin + docetaxel	30	15.0
(Cyclophosphamide/5-FU) + Epirubicin + docetaxel	35	17.5
Docetaxel or paclitaxel	21	10.5
Paclitaxel/docetaxel + carboplatin	8	4.0
Gemcitabine + docetaxel or Gemcitabine carboplatin	3	1.5
Capecitabine	6	3.0
Vinorelbine	2	1.0
**Hormonal therapy**		
Tamoxifen	31	15.5
Letrozole	29	14.5
Anastrozole	18	9.0
Tamoxifen or Letrozole + goserelin	3	1.5
**HER2-targeted therapy**		
Trastuzumab	9	4.5
Trastuzumab + tamoxifen or letrozole	5	2.5

### QLQ profile of breast cancer patients based on EORTC QLQ C-30 scales

Mean global health-QOL score of participants was 53.2 ± 1.9, with 54.0% of patients having poor global health-QOL status. The overall physical functioning scales was 67.8 ± 1.6, with 73.5% of patients had good physical functioning/QOL. Among the functional scales, cognitive functioning was the highest score (80.7 ± 1.8) while social functioning was the lowest one (57.8 ± 2.3). In QLQ-C-30, the mean symptoms scale was 36.0 ± 1.7, indicating that the majority of patients (71.5%) had good QOL, with low level of symptomatology. The most distressing symptoms were financial difficulties and insomnia (mean 63.7 ± 2.9 and 52.3 ± 3.1, respectively). Among symptom scales, dyspnea was the least distressing symptoms (mean 13.8 ± 1.8) (Table 4).

**Table 4:** QLQ profile of breast cancer patients based on EORTC QLQ C-30 scales

QOL domains	Mean ± SEM	Poor QOL N (%)	Good QOL N (%)
**Global health status**	**53.2 ± 1.9**	**108 (54.0%)**	**92 (46%)**
**Functional scales**			
Physical functioning	72.7 ± 1.6	29 (14.5%)	171 (85.5%)
Role functioning	62.6 ± 2.7	75 (37.5%)	125 (62.5%)
Emotional functioning	63.0 ± 2.6	74 (37.0%)	126 (63.0%)
Cognitive functioning	80.7 ± 1.8	40 (20.0%)	160 (80.0%)
Social functioning	57.8 ± 2.3	102 (51.0%)	98 (49.0%)
Total	67.8 ± 1.6	53 (26.5%)	147 (73.5%)
**Symptom scales**		**Poor QOL**	**Good QOL**
Fatigue	38.5 ± 2.4	66 (33.0%)	134 (67.0%)
Nausea/vomiting	33.3 ± 2.5	50 (25.0%)	150 (75.0%)
Pain	36.0 ± 2.4	55 (27.5%)	145 (72.5%)
Dyspnea	13.8 ± 1.8	25 (12.5%)	175 (87.5%)
Insomnia	52.3 ± 3.1	114 (57.0%)	86 (43.0%)
Appetite loss	37.2 ± 2.9	77 (38.5%)	123 (61.5%)
Constipation	20.7 ± 2.6	40 (20.0%)	160 (80.0%)
Diarrhea	27.2 ± 2.7	57 (28.5%)	143 (71.5%)
Financial difficulties	63.7 ± 2.9	132 (66.0%)	68 (34.0%)
Total	36.0 ± 1.7	57 (28.5%)	143 (71.5%)

### QLQ profile of breast cancer patients based on QLQ-BR-23

Based on QLQ-BR-23 scales, future perspective scored the highest functioning (74.7 ± 2.5), with 74.5% of patients having good QOL. On the contrary, worse functioning was found in body image (mean 47.7 ± 2.7), with 54.5% of patients having poor QOL. In the symptom scale, “being upset by hair loss” was the worse functioning (62.1 ± 3.3), with 68.6% of patients having poor QOL. Breast symptoms represent the low level of symptomatology (26.7 ± 1.5), with good level of QOL (87.0%) among breast cancer patients (Table 5).

**Table 5:** QLQ profile of breast cancer patients based on QLQ-BR-23

Variables	Mean ± SEM	Poor QOL N (%)	Good QOL N (%)
**Functional scales**			
Body image	47.7 ± 2.7	109 (54.5%)	91 (45.5%)
Future perspective	74.7 ± 2.5	51 (25.5%)	149 (74.5%)
**Symptom scales**			
Systemic therapy side effects	34.9 ± 1.8	53 (26.5%)	147 (73.5%)
Breast symptoms	26.7 ± 1.5	26 (13.0%)	174 (87.0%)
Arm symptoms	29.3 ± 1.8	47 (23.5%)	153 (76.5%)
Upset by hair loss (*n *= 118)	62.1 ± 3.3	81 (68.6%)	37 (31.4%)

### Impact of different medications on QLQ of breast cancer patients based on EORTC QLQ C-30 scales

Based on global health status of EORTC QLQ C-30, QOL was significantly higher in patients taking HER2-targeted therapy or hormonal therapy compared with conventional therapy (*P* = 0.000). In social functioning, role functioning and emotional functioning scales, patients taking HER2-targeted therapy or hormonal therapy had significant better QOL than patients taking conventional therapy (*P* = 0.000, *P* = 0.000 and *P* = 0.002, respectively) (Table 6). In symptoms scale, there was a marked significantly reduction in the level of symptomatology (fatigue, nausea/vomiting, pain, insomnia, appetite loss and diarrhea) in patients taking HER2-targeted therapy or hormonal therapy than those taking conventional chemotherapy (*P* = 0.000). Furthermore, financial difficulties scale was worse in patient taking conventional chemotherapy than those on HER2-targeted therapy (*P* = 0.049) (Table 6).

**Table 6:** Impact of different medications on QLQ of breast cancer patients based on EORTC QLQ C-30 scales

EORTC QLQ C-30 scales	QOL			*P* value
	**Conventional chemotherapy**	**Hormonal therapy**	**Targeted therapy**	
**Global health status**	**42.60 ± 2.5**	**64.76 ± 2.5**	**67.23 ± 7.1**	0.000***
**Functional scales**				
Physical functioning	71.17 ± 2.4	73.25 ± 2.3	80.95 ± 4.9	0.32
Role functioning	48.89 ± 3.9	75.93 ± 3.3	88.10 ± 7.0	0.000***
Emotional functioning	54.36 ± 3.9	73.46 ± 3.5	69.06 ± 8.3	0.002**
Cognitive functioning	82.70 ± 2.6	77.99 ± 2.6	80.96 ± 7.5	0.476
Social functioning	46.67 ± 3.1	68.73 ± 3.3	78.57 ± 7.8	0.000***
**Symptom scales**				
Fatigue	52.39 ± 3.3	24.41 ± 2.7	15.87 ± 7.0	0.000***
Nausea/vomiting	53.65 ± 3.3	9.88 ± 2.3	16.67 ± 8.5	0.000***
Pain	46.99 ± 3.4	24.06 ± 3.0	21.42 ± 7.8	0.000***
Dyspnea	15.56 ± 2.8	12.75 ± 2.6	7.14 ± 5.1	0.484
Insomnia	65.08 ± 4.0	39.51 ± 4.7	30.96 ± 10.7	0.000***
Appetite loss	52.71 ± 4.1	21.40 ± 3.8	11.90 ± 7.4	0.000***
Constipation	23.49 ± 3.9	17.69 ± 3.7	16.66 ± 8.3	0.527
Diarrhea	40.00 ± 4.1	9.46 ± 2.4	33.34 ± 12.5	0.000***
**Financial difficulties**	**70.16 ± 3.9**	**55.14 ± 4.6**	**64.29 ± 10.7**	0.049*

### Impact of different medications on QLQ of breast cancer patients based on QLQ-BR-23 scales

In symptoms scale of QLQ-BR-23, a significant reduction in the level of symptomatology (systemic side effects, arm symptoms and being upset by hair loss) had been observed in patients taking hormonal or HER2-targeted therapies when compared with patients taking conventional chemotherapy (*P* = 0.000 and *P* = 0.000 and *P* = 001, respectively) (Table 7).

**Table 7:** Impact of different medications on QLQ of breast cancer patients based on QLQ-BR-23 scales

QLQ-BR-23 scales	QOL			*P* value
	**Conventional therapy**	**Hormonal therapy**	**Targeted therapy**	
**Functional scales**				
Body image	47.85 ± 3.9	47.94 ± 4.0	45.24 ± 9.5	0.97
Future perspective	72.70 ± 3.7	75.31 ± 3.6	85.72 ± 8.3	0.436
**Symptom scales**				
Systemic therapy side effects	49.22 ± 2.3	17.75 ± 1.7	26.53 ± 5.7	0.000***
Breast symptoms	26.27 ± 2.2	28.09 ± 2.4	21.43 ± 4.0	0.568
Arm symptoms	17.88 ± 2.0	41.57 ± 2.7	43.66 ± 7.4	0.000***
Upset by hair loss	67.72 ± 3.5	37.49 ± 8.5	33.34 ± 14.9	0.001**

^**^P ≤ 0.01; ***P ≤ 0.001

### Factors affecting quality of life of breast cancer patients in Khartoum Oncology Hospital

The predictors of good QOL of BC patients were evaluated using logistic regression. In QLQ-C30 functional scales, hormonal (S.E = 0.53, OR = 3.7, *p* = 0.01) and HER2-targeted (S.E = 1.13, OR = 10.2, *p* = 0.04) therapies were positive predictors of good QOL in BC patients. According to the odds ratio, patients taking hormonal therapy (OR = 3.7, 95% CI = 1.32–10.63) were almost 4 times more likely to have good QOL than patients taking conventional chemotherapy. Moreover, patients on HER2-targeted therapy (OR = 10.2, 95% CI = 1.11–93.88) had 10.2 times chances to be in a good QOL than patients taking conventional chemotherapy. In QLQ-C30 symptoms scale, patients on hormonal therapy (OR = 0.07, *p* = 0.000, 95% CI = 0.02–0.29) were less likely to experience worse symptoms than patients taking conventional chemotherapy. Although the relationship between HER2-targeted therapy and conventional therapy was non-significant (*p*=0.08), HER2-targeted therapy appears to reduce symptomology levels (OR=0.22)  (Table 8).

**Table 8:** Factors associated with quality of life of patients with breast cancer at Khartoum Oncology Hospital

Variables	QLQ-C30-Functional scales			
	**S.E**	**Sig**	**OR**	**95% CI**
**Age**	0.94	0.135	0.25	0.04–1.55
**Comorbidity**	0.44	0.355	0.67	0.28–1.58
**Medications (ref. conventional therapy)**				
**Hormonal therapy**	0.53	0.01**	3.74	1.32–10.63
**Targeted therapy**	1.13	0.04*	10.2	1.11–93.88
**Stage of tumor (ref. stage 1)**				
**Stage 2**	0.73	0.13	0.33	0.08–1.39
**Stage 3**	0.41	0.26	0.64	0.29–1.41
**Cycle of chemotherapy**	0.41	0.36	0.69	0.31–1.54
	**QLQ-C30-Symptom scales**			
**Age**	1.01	0.516	1.921	0.27–13.79
**Comorbidity**	0.46	0.863	0.924	0.38–2.26
**Medications (ref. conventional therapy)**				
**Hormonal therapy**	0.67	0.000***	0.07	0.02–0.29
**Targeted therapy**	0.86	0.08	0.22	0.04–1.25
**Stage of tumor (ref. stage 1)**				
**Stage 2**	0.85	0.14	3.52	0.67–18.61
**Stage 3**	0.39	0.76	1.13	0.53–2.42
**Cycle of chemotherapy**	0.40	0.87	0.94	0.43–2.05

*S.E* Standard error, *OR* Odd ratio, *CI* Confidence interval

^*^P ≤ 0.05; **P ≤ 0.01; ***P ≤ 0.001

## Discussion

Breast cancer (BC) has surpassed lung cancer as the most commonly diagnosed cancer worldwide [1]. Cancer is the third leading cause of death, after malaria and viral pneumonia among Sudanese women [5]. Chemotherapy-related toxicity deteriorates the QOL of patients [24]. Therefore, QOL has become an important outcome measure in recent clinical trials [25, 26]. This study investigated the impact of BC medications on the QOL of BC patients in Khartoum oncology hospital.

In this study, the mean age of patients was 50 ± 11.7 years. This finding is in line with previous studies which reported that most BC patients were above 45 or 50 years old, and the disease frequently occurs in women around menopause [27–31]. However, BC can strike at any age; and 4% of cases occur in women under 40 years old [32].

The study results showed that most patients underwent modified radical mastectomy, and few patients (8.9%) had breast conserving surgery. This finding could be attributed to delay in detection and diagnosis of BC in Sudan as a result of unavailability and unaffordability of laboratory investigations (e.g., SLNB). In addition, lack of awareness about BC and delay in presentation may contribute in late detection of BC.

Regarding the systemic therapies, most patients were on conventional therapy, some patients were on hormonal therapy (tamoxifen and aromatase inhibitors) and few patients were on HER2-targeted therapy. Half of the patients received neoadjuvant therapy for locally advanced BC. Selection of systemic therapy is based on clinicopathological features and disease burden, in addition to the age, general health as well as the financial status of the patients. Conventional chemotherapy (cyclophosphamide/5-FU plus doxorubicin or epirubicin) is generally recommended for patients at high risk of recurrence. Others multiple-chemotherapy regimens are available worldwide. In the United States, doxorubicin and cyclophosphamide followed by paclitaxel (AC-T) is the frequently used regimen [12]. In this study, few patients received HER2-targeted therapy (trastuzumab) and most patients were on conventional chemotherapy; this could be attributed to inadequate availability and non-affordability of the targeted therapies in Sudan. In addition, it is not a routine practice in Sudan to assessments gene testing because of the unavailability and un-affordability of the tests. Because of financial constraints in most Sudanese patients, physicians usually prescribe conventional therapy to the patients regardless of the clinical benefit selectively towards HER2-targeted therapy or hormonal therapy. Consequently, financial hardship may adversely impact the health status and QOL of BC patients.

In terms of QLQ-C30 scale, the mean global health score was (53.2 ± 1.9), with more than fifty percent of patients had poor global health-QOL status. According to Chen et al. study, global health-QOL status was 53.8 ± 14.7 among Chinese cancer patients [27]. Slightly superior results about the global health status were achieved in preceding studies in Sudan (67 ± 17.8) and Bahrain (63.9 ± 21.3) [29, 33]. These discrepancies in results could be due to variation in the cancer stage and treatment modalities. In comparison with Bahraini women, 30% and 51.3% of patients had grade 0–1 and underwent lumpectomy, respectively, in Bahrain compared to this study (7% and 8.9% had grade 1 and lumpectomy, respectively). Moreover, most Bahraini women (92.8%) were non-metastatic patients who displayed good QOL. In addition, most Sudanese patients received conventional chemotherapy, and might experience chemotherapy-related toxicities that negatively influence their QOL.

In the overall functional scale, most patients (73.5%) had good QOL, with good cognitive functioning score (80.7 ± 1.8), and this could be due to relatively younger participants ( < 50 years). These findings are in line with study in Ethiopian BC patients with good cognitive functioning (80.06 ± 22.9) [28]. In the QLQ-C30 scales, the most distressing issue was the financial difficulties (63.7 ± 2.9); this may be due to the fact that Sudan is one of developing countries, with low economic status. This result is in accordance with previous studies in Ethiopia and Sudan where majority of patients had faced financial difficulties [28, 29]. Insomnia and fatigue were among the disturbing symptoms in this study, and these findings are consistent with earlier studies [33–35].

In terms of QLQ-BR23 functional scale, the future perspective scored the highest level. This may be mainly attributed to the patient’s family supports, in addition to their beliefs that everything in life happens according to God's will. According to the literature, more severe impairment was observed in future perspective among Chinese women, and this could be due to Chinese women with BC don’t want to burden their families with their deteriorating health [27]. The study results showed that worse functioning was found in body image (47.7 ± 2.7), with 54.5% of patients having poor QOL. This could be attributed to the fact that most patients underwent modified radical mastectomy (MRM) and lost their entire breast; and this could affect the woman's identity and remind her that a valuable part is missing from her feminized character. On the contrary, a study conducted in Bahrain reported that body image scored the highest level of functioning because the majority of patients underwent lumpectomy resulting in good body image [33]. In QLQ-BR23 symptoms scale, it was observed that “being upset by hair loss” was the highest disturbing symptom (62.1 ± 3.3), with the majority of patients having poor QOL. This can be attributed to the fact that hair is an essential part of a woman’s sexuality and gender identity, and any hair loss generates feelings of low self-esteem and anxiety from a perception of diminished attractiveness [36]. The results highlighted that the “systemic therapy side effects” was one of the distressing symptoms. As reported in literature, the majority of patients taking chemotherapy experienced systemic side effects that negatively affected their QOL [27, 33, 37, 38]. On the other hand, breast symptoms represent the low level of symptomatology (26.7 ± 1.5), with good level of QOL among breast cancer patients. The present finding is in line with Imran et al. study [38].

Regarding the impact of medications on patients’ QOL based on QLQ C-30 scale, the global health status was significantly higher in patients taking HER2-targeted therapy or hormonal therapy compared with conventional therapy. In social functioning, role functioning and emotional functioning scales, patients taking hormonal therapy or HER2-targeted therapy had significant better QOL than patients taking conventional therapy. A similar pattern of results was demonstrated in a previous study; global health status was declined after conventional chemotherapy. Likewise, cognitive functioning, emotional functioning, social functioning, role functioning, and physical functioning of the patients were significantly deteriorated during the first two cycles of chemotherapy [39]. Akin, et al.reported that all QOL dimensions were negatively affected in Turkish BC patients undergoing chemotherapy [40]. According to Tiezzi, et al.study, women treated with conventional chemotherapy experienced worse QOL in the physical functioning domains [41]. In QLQ C-30 symptoms scale, a marked significant reduction in the level of symptomatology (fatigue, nausea/vomiting, pain, insomnia, appetite loss and diarrhea) was observed in patients taking HER2-targeted therapy or hormonal therapy. Moreover, financial difficulties were significantly worse in patient taking conventional chemotherapy than those on HER2-targeted therapy.

In QLQ-BR-23 symptoms scale, a marked significant level of symptomatology (systemic side effects, arm symptoms and being upset by hair loss) was observed in patients taking conventional chemotherapy than patients on hormonal or HER2-targeted therapies. Greater toxicity of chemotherapy in comparison with hormonal or HER2-targeted therapy was reported in the literature data [42]. Osoba et al*.*, demonstrated that trastuzumab does not adversely affect QOL of patients [43]. A previous study showed that the toxicity profile of tamoxifen and aromatase inhibitors did not show significant reduction in the overall QOL [44]. Gadisa et al.reported that QOL of patients with BC was adversely affected by chemotherapy’s side effects [39].

Regarding the predictors of good QOL in BC patients, hormonal or HER2-targeted therapies were positive predictors of good QOL and less symptomology. Patients taking hormonal therapy were about 4 times more likely to have good QOL than those taking conventional chemotherapy. Moreover, patients on HER2-targeted therapy had 10.2 times chances to get good QOL than patients on conventional chemotherapy. A previous study demonstrated that systemic side effects of conventional chemotherapy (oral mucositis, constipation, peripheral neuropathy, anemia arthralgia/myalgia, dry mouth, diarrhea, constipation, and skin hyperpigmentation) were predictors for deteriorated QLQ [39].

Measuring QOL in BC patients, using validated disease-specific QoL measures, may help in improving patient’s well-being. Self-reported health status of patients can provide unique information leading to modifications in treatment plans and enhancing clinical care.

### Limitations

The cross-sectional design of the study (single center survey) and the relatively small sample size may lower the level of generalization of the research.

The study was conducted during COVID-19 pandemic; therefore, large sample size was not attained.

## Conclusion

This study concluded that BC survivors in Sudan had poor overall global health status, and financial difficulties. Insomnia, fatigue, poor appetite and upset by hair loss were the most distressing symptoms. Patients taking HER2-targeted or hormonal therapy scored significantly higher QOL than those on conventional therapy. Patients taking HER2-targeted therapy or hormonal therapy showed a marked significant reduction in the level of symptomatology (fatigue, nausea/vomiting, pain, insomnia, appetite loss, diarrhea, systemic side effects, arm symptoms and being upset by hair loss) than those on conventional therapy. Hormonal and HER2-targeted therapies were positive predictors of good QOL.

### Recommendations

Several effective interventions such as physical activity and psychosocial interventions proved to be effective in improving the QOL of BC patients. Therefore, integration of the palliative and supportive care (e.g., clinical treatments for symptoms, counseling, providing social, financial support and exercise) into the patients’ treatment program is urgently needed to improve their QOL.

Follow-up studies, assessing the pattern of QOL during and after the course of chemotherapy, are in demand to improve medication adherence and treatment plans.

## Supplementary Information

**Additional file 1.** 

## Data Availability

All data generated or analyzed during this study are included in this article [and its supplementary information files].
